# Self-care behavior and associated factors among adult heart failure patients in outpatient cardiac follow-up unit at Wachemo University Nigist Eleni Comprehensive Specialized Hospital, Southern Ethiopia

**DOI:** 10.1186/s12872-024-03901-3

**Published:** 2024-05-07

**Authors:** Ermias Sigebo Sugebo, Teshager Worku Kassie, Tesfaye Gobena, Temesgen Kechine Tibore, Sisay Foga Sebro, Tadesse Lelago Ermolo

**Affiliations:** 1 Department of Nursing, Wachemo University College of Medicine and Health Science, Hosaena, Southern Ethiopia; 2 Department of Nursing, Haramaya University College of Health and Medical Science, Harar, Ethiopia; 3 Department of Public Health, Haramaya University College of Health and Medical Science, Harar, Ethiopia

**Keywords:** Heart failure, Adult patients, Self-care behavior, Ethiopia

## Abstract

**Background:**

Heart failure is a serious medical condition that occurs when the heart is unable to pump sufficient blood to meet the needs of the tissues. Good self-care is an essential behavior in long term management and maintenance of physiologic stability, better medical and person-centered outcomes. Poor self-care behavior deteriorates the outcomes of heart failure patients. However, there were no sufficient evidences that illustrate the topic in the country, including the study area.

**Methodology:**

Institutional based cross-sectional study was conducted among 250 heart failure patients from July 5-August 4, 2021. All adult heart failure patients who fulfill the inclusion criteria and have appointment during study period were included in the study. Interview and medical chart review was used to collect data. Epidata version 3.1 and SPSS version 20 were used for data entry and analysis respectively. Bivariate and multivariable analysis was computed. The model fitness was checked by Hosmer and Lemeshow test.

**Results:**

From the total patients, 240 were interviewed with the response rate of 96%. Among these, 140(58.3%) [95% CI: 52.6, 64.9] had poor self-care behavior. Age>54: 9.891 [2.228, 43.922], poor knowledge: 6.980[1.065, 45.727], depression: 4.973[1.107, 22.338], low social support: 6.060[1.373, 26.739], insomnia: 4.801[1.019, 22.622] and duration with heart failure <1 year: 5.782[1.438, 23.247] were factors associated with poor self-care behavior.

**Conclusion:**

In this study, more than half of participants attending at Wachemo University Nigist Eleni Comprehensive Specialized Hospital in outpatient cardiac follow-up unit had poor self-care behavior. Of the study variables, older age, poor knowledge, depressive symptoms, low social support, insomnia and short duration with heart failure were related with poor self-care behavior. Thus, the findings highlight importance of assessing level of self-care behavior and implicate direction to take action to enhance level of self-care behavior.

## Introduction

Heart failure (HF) is a serious medical condition that occurs when the heart is unable to pump sufficient blood to meet the oxygen and nutrient needs of the tissues. This is due to a problem with contraction of the heart (systolic HF) or a problem with filling of the heart (diastolic HF) [1].The severity of symptoms and signs was classified into four classes (I-IV) by the New York Heart Association (NYHA) [1]. The Prevalence and burden associated with HF are progressively increasing in both developed and developing countries. Heart failure affects at least 26 million people globally [2], and in the US, approximately 6.2 million adults are living with heart failure, with an expected increase in the prevalence of 46% by 2030 [3]. In Africa, the cardiovascular diseases are the leading cause of morbidity and mortality, with an admission rate of up to 25% [4]. In the USA, the total cost of care (direct and indirect costs) for heart failure in 2020 is expected to reach $43.6 billion, with over 70% of costs attributed to medical costs [5]. During the 2015–2019 periods, total HF-associated costs in Spain reached 15,373 euros ($18405.32) per person [6].

Worldwide, health care is focused on reducing re-hospitalization due to heart failure and improving survival and well-being [7]. Multidisciplinary management programs focused on enhancing HF self-care significantly reduce HF and all-cause of hospitalizations in patients with heart failure [8].Self-care behavior is a modifiable factor that emphasizes the action to be taken by HF patients to maintain life and healthy functioning and to improve overall health-related quality of life [9]. In this process, heart failure patients engage in their own care and make decisions about managing symptoms or illness by performing it at any time, even in healthy and ill states [10]. The key elements of self-care include three behaviors. First, self-care maintenance; second, self-care monitoring and third, self-care management [7, 10, 11]. Good self-care is an essential behavior in long-term management and maintenance of physiological stability and better medical and person-centered outcomes [12, 13]. Poor self-care behavior deteriorates the outcomes of heart failure patients. Self-care behavior among heart failure patients in both developed and developing countries such as Ethiopia is poor [14]. This leads patients to often experience poor quality of life, poor treatment outcomes and recurrent hospitalization with developing serous symptoms, including breathing problems, fluid retention with weight gain and leg edema, and chronic fatigue [15–17]. The majority of HF patients in Ethiopia develop mental disorders and depression (51.1%), due to poor self-care behavior [18]. Different studies in both developed and developing countries have shown a wide range of levels of poor self-care behavior. According to a secondary analysis conducted in Sweden, the percentage of poor self-care was 59% [19]; in Kenya, the percentage of poor self-care behavior was 50.8% [20]; and in Ethiopia, the percentage of poor self-care behavior was 62.3% in west Amhara Region Referral Hospitals (14), 62.7% in the Jimma University Specialized Hospital [21], and 54.2% in the Tigray region [22]. Factors associated with poor self-care behavior were New York Heart Association (NYHA) class, co morbidity, duration of disease, knowledge deficit, depressive symptoms, anxiety, age, gender, income, social support, educational levels, marital status, living status, smoking cigarette, alcohol consumption, insomnia and functional capacity. [22–27].

There were limited studies have been conducted on self-care behavior and associated factors among heart failure patients in our country [14, 22, 28]. Additionally, there was no sufficient evidence that illustrate the topic in the study area.

Therefore, this study was aimed to assess the level of self-care behavior and associated factors among adult heart failure patients at the outpatient cardiac follow-up unit of Wachemo University Nigist Eleni Comprehensive Specialized Hospital (WCUNECSH), Hosanna, and southern Ethiopia.

## Conceptual framework

**Fig. 1:**
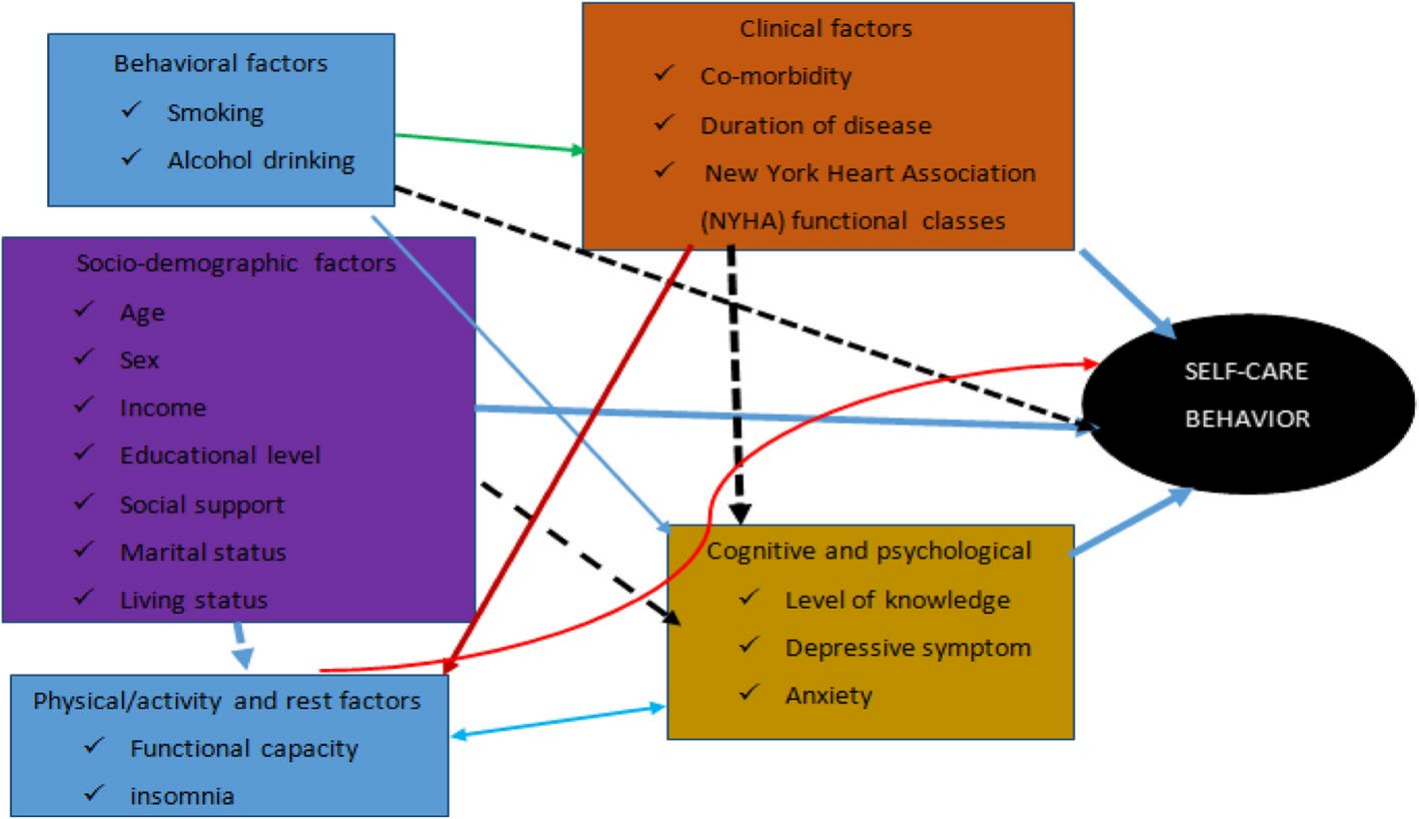
Conceptual framework for assessing self-care behavior and associated factors among adult heart failure patients in the outpatient cardiac follow-up unit at WCUNECSH, South Ethiopia, 2021

On the basis of previous scholarly articles, the conceptual framework for factors that are assumed to be associated with self-care behavior is categorized into four categories as shown in Fig. 1 socio-demographic, cognitive and psychological, clinical and behavioral factors.

## Materials and methods

### Study setting and period

The study was conducted at Wachemo University Nigist Elleni Comprehensive Specialized Hospital (WCUNECSH) in the Hadiya Zone, Hossana, from July 5-August 4, 2021. Hosanna Town is one of the largest towns of the Southern Nation Nationalities and People Region (SNNPR), which is the capital of the Hadiya Zone. It is found 230 km far from Addis Ababa and 168 km from Hawassa, the capital city of the SNNPR. In the zone, there are sixty-five health centers, three district hospitals and one comprehensive specialized hospital, Wachemo University Nigist Elleni Comprehensive Specialized Hospital (WCUNECSH). The WCUNECSH has different specialty clinics providing health care services. Among these, the cardiac follow-up clinic is a unit that serves 250 adult heart failure patients per month.

### Study design

An institutional based cross-sectional study was conducted.

### Source population

All adult patients who had a confirmed diagnosis of heart failure by clinical practitioners and who had regular follow-up at outpatient cardiac units.

### Study population

All adult patients with heart failure who were eligible based on the inclusion criteria and had appointments within the study period in outpatient cardiac follow-up units were eligible.

### Inclusion criteria

All adult heart failure patients were diagnosed by clinical practitioners and underwent regular follow-up at the outpatient cardiac unit during the study period.

### Exclusion criteria

Heart failure patients who were critically ill, unable to hear and unable to participate in the study were excluded.

### Sample size determination

The sample size was determined based on the single population proportion formula for objective one by using assumption of 95% confidence interval, good self-care behavior prevalence (45.8%) from a study conducted in Tigray [22], a 5% margin of error and for associated factors, as shown in Table 1. The calculated sample sizes for objectives one and two were 381 and 2222, respectively. Then, to have a possible maximum sample size, a larger number of samples were obtained from the initial samples calculated by objective two (2222) was taken. Since the source population of the study was less than 10,000, the finite population correction formula was used. The final sample size calculated by using associated factors and considering a 10% non-response rate was 248.

**Table 1:** Sample size determination by using the Epi Info statistical package version 7.2.4.0 for different associated variables

Assumptions	Associated variables	AOR(95% CI)	%outcome on unexposed group	%outcome on exposed group	Initial Calculated sample	reference
Power=80% CI= 95% 1:1 Ratio	Age: ≥55,18-27	3.25(1.19–8.86)	23.5	31	1158	[22]
	Level of education: can’t read and write, Diploma and above	2.55(1.08–6.01)	15.5	34.2	188	
	NYHA class: class I, class IV	0.17(0.06–0.49)	16	11.8	2222	
	Co morbidity: yes, No	5.01(2.71–9.29)	55.1	44.9	792	
	Duration of HF: <1year, ≥1year	0.38(0.22–0.68)	27.3	72.7	44	
	Knowledge of HF: poor, good	6.49(3.52–11.94)	36.9	63.1	128	
	Social support: low, high	2.34(1.32–4.18)	26.2	73.8	42	

### Sampling Procedure and Sampling Technique

The total population attending the outpatient cardiac follow-up unit was 250 patients. All heart failure patients who fulfilled the inclusion criteria and had appointments during the study period were included as study subjects since the source population was almost equivalent to the final sample size. The sampling frame was a list of heart failure patients attending the outpatient cardiac follow-up units.

### Data collection tool

The data collection tool used was an interviewer-assisted structured questionnaire. The investigator prepared the questionnaire for face-to-face interviews with heart failure patients by adopting from different studies and validated sources. It had 10 parts:

- Part I: Included questionnaire used to assess socio-demographic characteristics such as age, sex, marital status, and living status, level of education, occupation, residency and income of participants.
- Part II: Modified European Heart Failure Self-Care Behavior Scale (EHFScBS), an 11-item questionnaire in which patients rated their self-care behavior on a 5-point Likert-type scale from 1 (I completely disagree) to 5 (I completely agree). In this scale, the total score ranges from 11 to 55 by summing the scores for each item [22, 28, 29]. The reliability of the EHFScBS was good (Cronbach’s alpha=.803).
- Part III: Questionnaire used to assess the knowledge of heart failure patients by using Japan’s Heart Failure Knowledge Scale (JHKS) with an 8-item questionnaire. The questionnaire has choices patients to respond “yes or no” or “I do not know” to each item. A correct answer was assigned a score of 1, and an incorrect answer (I do not know) was assigned a score of 0. Then, the scores for each item were summed, giving a range of total scores from 0 to 8 [14, 29].
- Part IV: Questionnaire used to assess the patient’s depression using patient health questionnaire 9 (PHQ-9) scales. This is a 9-item scale that measures depressive feelings and behaviors on a four-point Likert scale ranging from “Not at all (0)” to “Nearly every day (3)”. The scores for each item are summed to obtain a range of total scores from 0 to 27 [30, 31].
- Part V: Questionnaire used to assess patients’ anxiety using general anxiety disorder (GAD-7) Screening Tool. This is a 7-item scale that measures anxiety disorder on a 4-point Likert scale ranging from “Not at all (0)” to “Nearly every day (3)”. The scores for each item are summed to obtain a range of total scores from 0 to 21 [32].
- Part VI: Questionnaire used to assess social support using the Multidimensional Scale of Perceived Social Support (MSPSS). This 12-item scale is used to assess social support on a 7-point Likert scale ranging from “1 (very strongly disagree)” to “7 (very strongly agree)”. The mean score for each item was computed to obtain a total score ranging from 1 to 7 [22, 33].
- Part VII: questionnaire used to assess insomnia using Insomnia Severity Index (ISI). This is 7-item scale is used to assess insomnia in the last two weeks on a five-point Likert-type scale ranging from 0 (none/very satisfied/not at all noticeable/not at all worried/not at all interfering) to 4 (very severe/very dissatisfied/very much noticeable/very much worried/very much interfering). Its score is calculated by summing results, which ranges from 0 to a maximum 28 [34].
- Part VIII: The Duke Activity Status Index (DASI) is a self-administered questionnaire that measures a patient's functional capacity and includes 12 activities representative of major aspects of physical function. A score is calculated based on weighted answers from 12 questions related to daily activities of living and then summed to form individual patient DASI scores ranging from 0 (all “no” answers) to 58.2 (all “yes” answers) [35, 36].
- Part IX: Questionnaire used to assess substance use. This is a four-item questionnaire with two questions for alcohol use assessment and two questions for cigarette smoking assessment [37, 38].
- Part X: Medical chart review used to identify clinical profiles of patients from patients’ medical records. This is a three-item questionnaire to assess the NYHA functional class, co morbidity status and duration of disease.

### Data collection procedures

All participants who fulfilled the inclusion criteria were included. Data were collected from patients via interviews using interviewer-assisted structured questionnaires prepared for those who provided informed consent and who reviewed patients’ record for co morbidity status, duration of disease and NYHA functional class.

## Operational definition

### Good self-care behavior

Heart failure patients who scored ≥44 on total modified European Heart Failure Self-Care Behavior Scale (EHFScBS) scores ranging from 11 to 55 points [22, 28, 29].

### Poor self-care behavior

Heart failure patients who scored <44 points on total scores ranging from 11 to 55 points on the modified European Heart Failure Self-Care Behavior Scale (EHFScBS) [22, 28, 29].

### Patients with good knowledgeable

Heart failure patients who correctly answered ≥75% (≥6) of the knowledge related questions of Japan’s Heart Failure Knowledge Scale, with scores ranging from 1 to 8 [14, 29].

### Patients with poor knowledge

Heart failure patients who answered <75% (<6) of knowledge related questions of Japan’s Heart Failure Knowledge Scale with scores ranging from 1 to 8 [14, 29].

### Depressed

Patients who scored ≥10 on depression-related questions ranging from 0 to 27 [30, 31].

### Anxiety

Heart failure patients who scored ≥10 on anxiety screening scale scores ranging from 0 to 21 [32].

### High social support

Heart failure patients who score above the mean on the Multidimensional Scale of Perceived Social Support (MSPSS) score ranges from 1 to 7 [22, 33].

### Insomnia

Heart failure patients who scored ≥8 points on Insomnia severity index, which ranges from 0 to28 [34].

### No Insomnia

Heart failure patients who score 0-7 points out of 28 points on the Insomnia Severity Index which ranges from 0 to28 points [34].

### Good functional capacity

Heart failure patients with total DASI scores ≥31.95 ranges from 0 to 58.2 [36].

### Poor functional capacity

Heart failure patients with total DASI scores <31.95 ranges from 0 to 58.2 [36].

### Co morbidities

Hypertension, diabetes mellitus, kidney disease and others [39].

### Current users of substance

When the respondents used a specified substance (alcohol or cigarette) for nonmedical purposes in the last three months [37, 38].

### Ever users of substance

When the respondents use a specified substance (alcohol or cigarette) for nonmedical purposes even once in their lifetime [37, 38].

## Data quality control

To ensure the quality of the data, the questionnaire was examined by senior experts for content validity, and a pretest was performed on 5% of the total population. To maintain consistency of the tool, the questionnaire was first translated from English to Amharic and Hadiyyisa. Then, the Amharic and Hadiyyisa versions of the questionnaire were translated back to English by a language expert who was blinded to the first English version of the questionnaire. The principal investigator provided training to the data collectors and supervisor for two days before the actual data collection task. The data were collected by two BSc nurses and Supervisor who closely followed and supervised the data collectors. All completed questionnaires were examined for completeness and consistency during data management, storage, cleaning and analysis by the principal investigator. To minimize the recycling of the participants, the card was checked, and color code was given for each patient card.

### Data processing and analysis

After the collected data were checked for its consistency and completeness, they were cleaned, coded, checked, and entered into Epidata version 3.1, after which they exported to the Statistical Package for the Social Sciences (SPSS) version 20 for analysis. Bivariate logistic regression was computed to identify the direction, magnitude and strength of the associations between the outcome variable and explanatory variables. To control for confounding effects, those variables with a p-value ≤0.25 significance level in the bivariate logistic regression were entered into the multivariable logistic regression model. Then, variables with *p*-value <0.05 in the multivariable logistic regression analysis were considered as significantly associated variables. Adjusted odds ratios with 95% confidence intervals were calculated to measure the strength of associations and to determine statistical significance. The model fitness was checked by the Hosmer and Lemeshow goodness of fit test in the logistic regression model (*p*>0.05). The Multicollinearity was checked with the variance inflation factor (VIF). Variables with a VIF>10 were excluded from the multivariate analysis.

**Ethical considerations**

An ethical clearance and approval letter was obtained from the Institutional Health Research Ethics Review Committee (IHRERC) College of Health and Medical Sciences of Haramaya University after review for the absence of any harm to human subjects. Written permission to conduct the study in the cardiac follow-up unit was obtained from the WCUNECSH concerning the body. After the purpose and possible benefit of the study were explained, informed voluntary written and signed consent was obtained from each patient who agreed to participate before starting the data collection procedure. Informed voluntary written and signed consent for participants who were unable to read and write was obtained from their legal guardians.

## Results

### Socio-demographic characteristics

Of the 250 heart failure patients included in the study, 240 were eligible for interviews, for a response rate of 96%. Of the 240 respondents, 86 (35.8%) were >54 years old. One hundred thirty-five (56.3%) of the respondents were females. Among the participants, 120 (50.0%) were married, and 103 (42.9%) were single. Most of the participants (152, 63.3%) lived with family. Of the total of respondents who participated in the study, 98 (40.8%) were unable to read and write. Of the participants, 53 (22.1%) were housewives. More than half of the respondents involved in the study (138, 57.5%) were rural dwellers. Nearly two-thirds (61.7%) of the respondents had received ≥586 Ethiopian Birr per month. Of the 240 participants, 94 (39.2%) had low social support, with a mean ± standard deviation of 4.3±1.4 for multi-dimensional social support score (Table 2)

**Table 2:** Socio-demographic characteristics of adult heart failure patients in the outpatient cardiac follow-up unit at Wachemo University Nigist Eleni Comprehensive Specialized Hospital, Hosanna, and Southern Ethiopia 2021

**Variables**	**Categories**	**Frequencies (*N*=240)**	**%**
Age	>54	86	35.8
	≤54	154	64.2
Sex	Male	105	43.8
	Female	135	56.3
Marital status	Single	103	42.9
	Married	120	50.0
	Divorced	17	7.1
Living status	Alone	88	36.7
	With family	152	63.3
Level of education	Cannot read and write	98	40.8
	Primary school	32	13.3
	Secondary school	31	12.9
	Diploma and above	79	32.9
Occupation	Farmer	26	10.8
	Housewife	53	22.1
	Merchant	41	17.1
	Government employee	30	12.5
	Others ^a^	90	37.5
Residency	Urban	102	42.5
	Rural	138	57.5
Income	<586	92	38.3
	≥586	148	61.7
Social support	Low	94	39.2
	High	146	60.8

^a^Beggar, pensioner

### The level of Self-care Behavior

**Fig. 2:**
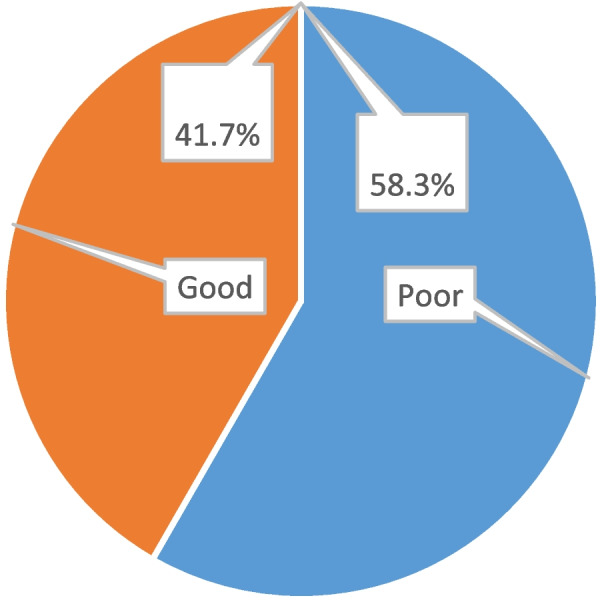
Self-care behavior among adult heart failure patients in the outpatient cardiac follow-up unit at Wachemo University Nigist Eleni Comprehensive Specialized Hospital, Hosanna Southern Ethiopia, 2021

The level of self-care behavior (SCB) was measured by modified European heart failure self-care behavior scale (EHFScBS), with 11-item questionnaire, which have 5 point likert scale 1’I completely disagree’ to 5’I completely agree.’ Then, categorized into ‘good SCB’ for those scored 44 to 55 and ‘poor SCB’ for those scored 11 to 43. As shown in Fig. 2, more than half (58.3%, 95% CI: 52.6, 64.9) of the participants had poor self-care behavior. The poorest self-care behaviors were related to poor practice of regular physical exercise (106, 44.2%), daily weight monitoring (98, 40.8%) and taking shortness of breath as easy (96, 40.0%). The mean heart failure self-care behavior score was 39.1±6.8.

### Cognitive and psychological factors

Among the 240 eligible participants, (*n*=143; 59.6%) had poor knowledge about self-care behavior (Table 3). The mean heart failure knowledge score was 4.9±2.1. The minimum and maximum responses of the study subjects, with knowledge scale scores ranging from 0 to 8, were 1 and 8, respectively. The major areas where poor knowledge was identified were knowledge about heart failure (*n*=130; 54.2%), identifying symptoms of lung congestion with fluid (*n*=148; 61.7%) and knowledge about diuretic action (*n*=144; 61.7%).

**Table 3:** Cognitive and psychological factors among adult heart failure patients in the outpatient cardiac follow-up unit at Wachemo University Nigist Eleni Comprehensive Specialized Hospital, Hosanna Southern Ethiopia 2021

**Variables**	**Categories**	**Frequencies (*N*=240)**	**%**
Level of knowledge	Poor	143	59.6
	Good	97	40.4
Depression	Yes	146	60.8
	No	94	39.2
Anxiety	Yes	141	58.8
	No	99	41.2

Approximately two-third of respondents (146, 60.8%) had depressive symptoms, with a mean score of 10.9±4.6 on the PHQ-9 brief depression severity measurement scale (Table 3). The minimum and maximum responses of depressive symptoms among respondents in the range of 0 to 27 were 0 and 22, respectively.

One hundred forty-one (58.8%) of the participants had anxiety, with a mean score of 9.6±4.2 on the General Anxiety Disorder Scale (GAD-7) and Severity Screening and measurement scale, whereas the minimum and maximum scores on the Anxiety Measure scale ranged from 0 to 21 were 0 and 19, respectively.

### Physical/ activity and rest/ factors

**Fig. 3:**
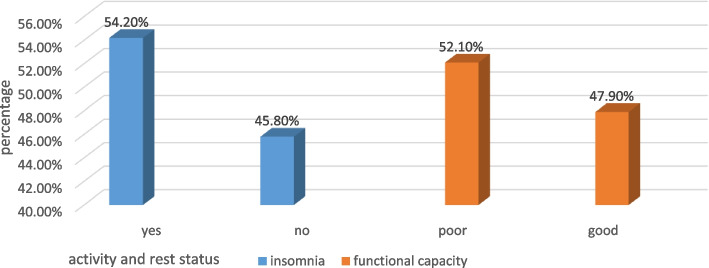
Activity and rest factors of adult heart failure patients in the outpatient cardiac follow-up unit at Wachemo University Nigist Eleni Comprehensive Specialized Hospital, Hosanna Southern Ethiopia 2021

More than half of the study subjects (54.2%) had insomnia, while 110 (45.8%) did not have insomnia with range of response on the insomnia severity index scores between 1 and 28. The mean insomnia severity index score was 10.3±5.0 (Fig. 3).

Of the 240 respondents, 125 (52.1%) had poor functional capacity for performing physical activity, with minimum and maximum ranges of response for activity status index scores between 0 and 50.7, respectively (Fig. 3).

### Behavioral and clinical factors

Of the 240 participants, 48 (20.0%) were alcohol drinkers. Among these participants, 30 (62.5%) were current drinkers, whereas 18 (37.5%) were ever drinkers of alcohol. Of the 240 study subjects, 31 (12.9%) were cigarette smokers. Among these, 17 (54.8%) were current smokers, whereas 140 (45.2%) were ever smokers (Table 4).

**Table 4:** Behavioral and clinical factors among adult heart failure patients in the outpatient cardiac follow-up unit at Wachemo University Nigist Eleni Comprehensive Specialized Hospital, Hosanna Southern Ethiopia 2021

**Variables**	**Categories**		**Frequencies (*N*=240)**	**%**
Alcohol drinking	Yes	within last three months	30	62.5
		before three months ago	18	37.5
	No		192	80.0
Cigarette smoking	Yes	within last three months	17	54.8
		before three months ago	14	45.2
	No		209	87.1
NYHA class	I		57	23.8
	II		37	15.4
	III		72	30.0
	IV		74	30.8
Co morbidity	Yes		130	54.2
	No		110	45.8
Duration with heart failure	<1 year		102	42.5
	≥1 year		138	57.5

Fifty-seven (23.8%) of the respondents had New York Heart Association (NYHA) class one, while 74 (30.8%) of the respondents had NYHA class four. More than half of the participants (54.2%) had clinically confirmed co morbidities. In addition, 138 (57.5%) of the respondents had heart failure for ≥1 year duration.

### Factors associated with self-care behavior among adult heart failure patients

According to bivariate logistic regression analysis, age, sex, marital status, living status, level of education, income, social support, level of knowledge, depression, anxiety, insomnia, New York Heart Association (NYHA) class, co morbidity and duration of heart failure were significantly associated with the outcome variable, with p*values ≤0.25. However, according to the multivariate logistic regression model shown in Table 5, only six of the candidate variables were significantly associated with self-care behavior, with p*values <0.05. According to the multivariate logistic regression analysis, age, level of knowledge, depression, low social support, insomnia and duration of heart failure were significantly associated with the outcome variable at the 95% confidence level, with a p*value <0.05 (Table 5).

**Table 5:** Bivariate and multivariable logistic regression analysis results among adult heart failure patients in the outpatient cardiac follow-up unit at Wachemo University Nigist Eleni Comprehensive Specialized Hospital, Hosanna Southern Ethiopia 2021

**Variables**	**Categories**	**Self-care behavior(SCB)**		**COR(95%CI)**	**AOR(95% CI)**	P*values
		Good N(%)	Poor N(%)			
Age	>54	28(32.6%)	58(67.4%)	1.819(1.048, 3.156)	9.891(2.228, 43.922)	**.003**
	≤54	72(46.8%)	82(53.2%)	1	1	
Sex	Female	49(36.3%)	86(63.7%)	1.658(.986, 2.786)	1.054(.303, 3.665)	.934
	Male	51(48.6%)	54(51.4%)	1	1	
Marital status	single	30(29.1%)	73(70.9%)	2.876(1.649, 5.017)	2.439(.507, 11.739)	.266
	divorced	5(29.4%)	12(70.6%)	2.836(.941, 8.549)	.400(.029, 5.579)	.496
	married	65(54.2%)	55(45.8%)	1	1	
Living status	Alone	16(18.2%)	72(81.8%)	5.559(2.963, 10.427)	2.832(.505, 15.889)	.237
	With family	84(55.3%)	68(44.7%)	1	1	
Level of education	Can’t read & write	8(8.2%)	90(91.8%)	35.526(14.614, 86.364)	2.215(.271, 18.131)	.458
	Primary school	11(34.4%)	21(65.6%)	6.029(2.467, 14.732)	1.568(.193, 12.763)	.674
	Secondary school	21(67.7%)	10(32.3%)	1.504(.604, 3.746)	1.545(.260, 9.162)	.632
	Diploma & above	60(75.9%)	19(24.1%)	1	1	
Income	<586	13(14.1%)	79(85.9%)	8.667(4.427, 16.967)	2.347(.546, 10.092)	.251
	≥586	87(58.8%)	61(41.2%)	1	1	
Level of knowledge	Poor	30(21.0%)	113(79.0)	9.765(5.363, 17.782)	6.980(1.065, 45.727)	**.043**
	Good	70(72.2%)	27(27.8%)	1	1	
Depression	Yes	20(13.7%)	126(86.3%)	36.0(17.207, 75.319)	4.973(1.107, 22.338)	**.036**
	No	80(85.1%)	14(14.9%)	1	1	
Anxiety	Yes	26(18.4%)	115(81.6%)	13.092(7.030, 24.383)	1.681(.444, 6.356)	.444
	No	74(74.7%)	25(25.3%)	1	1	
Social support	Low	10(10.6%)	84(89.4%)	13.500(6.469, 28.172)	6.060(1.373, 26.739)	**.017**
	High	90(61.6%)	56(38.4%)	1	1	
Insomnia	Yes	46(35.4%)	84(64.6%)	1.761(1.048, 2.958)	4.801(1.019, 22.622)	**.047**
	No	54(49.1%)	56(50.9%)	1	1	
NYHA class	I	5(8.8%)	52(91.2%)	10.978(3.940, 30.586)	4.361(.649, 29.320)	.130
	II	20(54.1%)	17(45.9%)	.897(.407, 1.979)	.418(.063, 2.761)	.365
	III	37(51.4%)	35(48.6%)	.998(.522, 1.911)	.453(.105, 1.961)	.290
	IV	38(51.4%)	36(48.6%)	1	1	
Co-morbidity	Yes	40(30.8%)	90(69.2%)	2.700(1.591, 4.581)	2.477(.573, 10.713)	.225
	No	60(54.5%)	50(45.5%)	1	1	
Duration with HF	<1 year	9(8.8%)	93(91.2%)	20.007(9.268, 43.188)	5.782(1.438, 23.247)	**.013**
	≥1 year	91(65.9%)	47(34.1%)	1	1	

Participants aged >54 years were 9.891 times more likely to have poor self-care behavior than participants aged ≤54 years [AOR: 9.891, 95% CI: 2.228, 43.922].

Heart failure patients who had poor knowledge about self-care behavior were 6.980 times more likely to have poor self-care behavior than those who had good knowledge [AOR: 6.980, 95% CI: 1.065, 45.727].

Respondents who had depressive symptoms were found 4.973 times more likely to have poor self-care behavior than respondents who had no depressive symptoms [AOR: 4.973, 95% CI: 1.107, 22.338].

Patients who had low social support were 6.060 times more likely to have poor self-care behavior than patients with high social support [AOR: 6.060, 95% CI: 1.373, 26.739].

Participants who had insomnia were 4.801 times more likely to have poor self-care behavior than were those who had no insomnia [AOR: 4.801, 95% CI: 1.019, 22.622].

Respondents who presented with a duration of heart failure <1 year were 5.782 times more likely to have poor self-care behavior than respondents whose duration of disease was ≥1 year [AOR: 5.782, 95% CI: 1.438, 23.247].

## Discussion

This study was aimed to assess the level of self-care behavior and associated factors among adult heart failure patients at the outpatient cardiac follow-up unit of Wachemo University Nigist Eleni Comprehensive Specialized Hospital (WCUNECSH), Hosanna, and southern Ethiopia. The findings of this study showed that, poor self-care behavior among heart failure patients was 58.3% (95% CI: 52.6, 64.9). This finding is consistent with studies conducted in Zimbabwe (53.8%) [40], Tigray (54.2%) [22], the western Amhara region (62.3%) [14] and Jimma University (59.2%) [28]. This similarity might be due to socioeconomic status of the countries where the studies were conducted. This finding is higher than those of studies conducted at Gondar Referral Hospital (48.0%) [16] and Kenya (50.8%) [20]. The possible variation might be due to differences in care-providing techniques, sample sizes and levels of health education for heart failure patients.

Regarding poor self-care behavior among heart failure patients, the poorest self-care behaviors observed in this study were on regular physical exercise (44.2%), daily weight monitoring (40.8%) and taking shortness of breath as easy (40.0%). This finding is in line with the findings of other studies conducted in the western Amhara region, which reported mean scores for regular physical exercise (4.62), daily weight monitoring (4.44) and taking shortness of breath as easy (4.30) [14] as well as Gondar Referral Hospital regular physical exercise (80.0%) and daily weight monitoring (91.6%) [39].

Concerning the factors associated with poor self-care behavior in this study, participants aged >54 years were 9.891 times more likely to have poor self-care behavior than participants aged ≤54 years. This was consistent with studies conducted in Jordan [41], Korea [17] and Tigray [22]. However, this finding is inconsistent with a study conducted at Jimma University Referral Hospital [28]. One possible explanation might be physiological changes in vision and fatigue related to age and disease conditions.

Heart failure patients who had poor knowledge about self-care behavior were 6.980 times more likely to have poor self-care behavior than those who had good knowledge. This result is supported by studies conducted in Tigray [22], the western Amhara region [14], Gondar Referral Hospital [39] and Jimma University referral hospital [28]. The possible reason might be that poor knowledge about disease may predispose heart failure patients to poor self-care behavior and give less attention to self-care activities.

Respondents who had depressive symptoms were 4.973 times more likely to have poor self-care behavior than respondents who had no depressive symptoms. This finding is supported by studies conducted at Jimma Referral Hospital [28] and in the western Amhara region [14]. This might be due to the decreased intention of patients with depression to follow recommended practices and perform self-care activities.

Patients who had low social support were 6.060 times more likely to have poor self-care behavior than patients with high social support. This finding is consistent with studies conducted in Korea [17] and Tigray [42]. This might be because social support can contribute to motivating patients to adopt and maintain up in all aspects of self-care activities [22].

Participants who had insomnia were 4.801 times more likely to have poor self-care behavior than were those who had no insomnia. This result is in line with a study conducted in the United States [24]. The possible reason might be that inadequate rest, stress related to poor sleep and altered quality of life reduces inspiration and attention to self-care behavior.

Respondents who presented with a duration of heart failure <1 year were 5.782 times more likely to have poor self-care behavior than respondents whose duration of disease was ≥1 year. This finding is supported by studies conducted in Tigray [22], the western Amhara region [14] and Jimma Referral Hospital [28]. The possible reason might be that experience and continued health education on self-care behavior for a long period of time can improve the level of awareness of the importance of self-care activities [22].

Limitations of this study is that the cause and effect relationships between outcome and different associated variables were not identified because of the nature of the study design (cross-sectional, which lacks follow-up evaluation). In addition, there may be information (recall/social desirability) biases because of the nature of the tool used (self-reporting). Therefore, researchers should conduct further studies with different designs such as prospective, follow-up, and qualitative research, in the same study area as well as in different parts of the country to further explore problems related to self-care behavior and to elucidate the relationships between factors and self-care behavior.

## Conclusion

Self-care behavior is crucial for heart failure patients to achieve positive and feasible health outcomes. In this study, more than half of the participants attending Wachemo University Nigist Eleni Comprehensive Specialized Hospital in the outpatient cardiac follow-up unit exhibited poor self-care behavior. Among the study variables, older age, poor knowledge, depressive symptoms, low social support, insomnia and short duration of heart failure were identified as factors associated with poor self-care behavior among heart failure patients. Thus, the findings highlight the importance of assessing the level of self-care behavior and implicate directions for taking action to enhance the level of self-care behavior. It is better if Wachemo University Nigist Eleni Comprehensive Specialized Hospital and governmental and private health institutions need to target patients with poor self-care behavior among adult heart failure patients to encourage patients to follow self-care strategies and link patients with depression and insomnia to psychiatric units for treatment and health profesionals to provide comperehensive counseling and health education to patients/families attending cardiac units.

## Data Availability

The datasets used and analyzed in the current study are included within the article.
